# Global research trends and hotspots in the health effects of Tai Chi on older adults: a bibliometric analysis (2010–2025)

**DOI:** 10.3389/fpubh.2026.1799937

**Published:** 2026-03-24

**Authors:** Yantao Niu, Qiushi Zhang, Qiaofang Liu, Min Wang

**Affiliations:** 1 Institute of Physical Education, Huaiyin Normal University, Huai’an, China; 2 Institute of Physical Education, Huzhou University, Huzhou, China; 3 School of Physical Education, Qufu Normal University, Qufu, China

**Keywords:** bibliometric analysis, healthy aging, older adults, research trends, Tai Chi

## Abstract

**Introduction:**

Tai Chi has potential benefits in promoting healthy aging, but a comprehensive understanding of its global research landscape, thematic evolution, and emerging trends remains limited. This study aims to use bibliometric methods to systematically analyze the knowledge structure, hotspot evolution, and collaborative networks of global research on Tai Chi and the health of the older adult(s) from 2010 to 2025, in order to explore future research hotspots and trends.

**Methods:**

A bibliometric approach was employed to conduct a visualized analysis of 2,532 eligible publications retrieved from the Web of Science (WOS) and Scopus databases. VOSviewer and CiteSpace software were used to examine publication trends, collaborative patterns, keyword co-occurrence, and thematic evolution.

**Results:**

The number of publications increased from approximately 80 in 2010 to a peak of about 270 in 2025, indicating that the field has entered a phase of accelerated development. China (1,034 publications, 41%) and the United States (865 publications, 34%) constituted the dual core of global research output. Institutions such as Shanghai University of Sport (52 publications) and Harvard University (38 publications) formed key cross-regional collaborative networks. Keyword co-occurrence analysis identified four major research clusters: balance and fall prevention (175 occurrences), quality of life (127 occurrences), depression (103 occurrences), and cognitive function. Burst keyword analysis revealed that the research frontier has shifted from “alternative medicine” toward “Parkinson’s disease” (burst strength = 3.66) and “network meta-analysis” (burst strength = 8.37).

**Conclusion:**

Research on Tai Chi has been deeply integrated into the modern rehabilitation medicine system, with the research paradigm shifting from single functional outcome validation toward multidimensional health promotion and precision evidence-based approaches encompassing physical, psychological, and cognitive domains. This study supports the inclusion of Tai Chi as a low-cost, high-efficiency mind–body intervention model within global strategies for promoting healthy aging.

## 1 Introduction

Global population aging represents one of the most significant public health challenges of the 21st century (1). The World Health Organization (WHO) estimates that by 2050, the population aged 60 and over will reach 2.1 billion, exceeding 22% of the global total (2). Sedentary behavior has been increasingly recognized as a critical risk factor for age-related functional decline and systemic inflammation (3). Promoting structured physical activity is essential not only for metabolic health but also for mitigating the psychosocial and physiological burdens of aging (4). The increased risks of falls, chronic diseases, cognitive decline, and mental health issues in this cohort have made effective, safe, and scalable methods for promoting health in older adults an international focus. Among numerous non-pharmacological intervention strategies, Tai Chi, characterized by its gentle, fluid, and holistic approach to mind and body, is widely used to improve physical function, cognitive abilities (2), and mental health in older adults. Due to its high safety profile, wide applicability, and ease of long-term adherence, it is currently among the most influential traditional Chinese exercises worldwide (5, 6).

Several studies have substantiated the benefits of Tai Chi in older adults, highlighting its multidimensional value in improving balance, preventing falls, and enhancing quality of life (7–10), and enhance lower limb muscle strength and joint flexibility (11, 12), and its positive effect on cardiopulmonary function (13), metabolic health (14), and chronic disease management (e.g., diabetes and hypertension) (15–17). Concurrently, Tai Chi’s physical and mental characteristics are effective in alleviating anxiety (18), depression, sleep disorders (19, 20), and cognitive decline (21). Recently, advances in neuroscience, sports rehabilitation, and public health have shifted academic focus toward the potential mechanisms underlying Tai Chi’s role in regulating brain function and emotional processing, and in improving executive function (22).

Despite the expansion of international research on Tai Chi and older adult(s) health, the field faces several limitations. First, the research landscape is highly fragmented, with significant disparities in research focus and contributions across different countries and regions. Second, there is a lack of systematic analyses adopting a global perspective on research topics, collaborative networks, knowledge structures, and cutting-edge trends. Third, while Tai Chi research is increasingly interdisciplinary, a clear knowledge map has not yet been established. To address these gaps, bibliometric analyses are needed to systematically reveal the field’s knowledge system.

While traditional systematic reviews and meta-analyses focus on quantifying the specific clinical efficacy of Tai Chi, bibliometric analysis provides a unique macro-perspective. It maps the evolution of intellectual structures, quantifies international collaboration networks, and identifies emerging research frontiers that traditional reviews may overlook. Bibliometric analysis utilizes mapping to visualize the clustering structure of research topics, the collaborative relationships among core authors and institutions, the evolutionary path of research hotspots, and the emergence of frontier topics (23). Visual bibliometric tools such as VOSviewer and CiteSpace have been widely used across international sports science, medicine, rehabilitation, and public health. However, a global, systematic bibliometric analysis specifically targeting research on Tai Chi and the health of older adults is currently lacking.

This study aims to address this gap by conducting a systematic bibliometric analysis of global research on Tai Chi and the health of older adults using the Web of Science and Scopus databases. By constructing a network of authors, institutions, and countries, identifying the co-occurrence structure of keywords, and analyzing the evolution of research hotspots and themes, we seek to answer the following questions: (1) What are the global publishing models and cooperation networks? (2) What are the core research themes and knowledge structures? (3) How do research hotspots and cutting-edge fields evolve over time?

## 2 Methods

### 2.1 Literature sources and data retrieval

**Figure 1:**
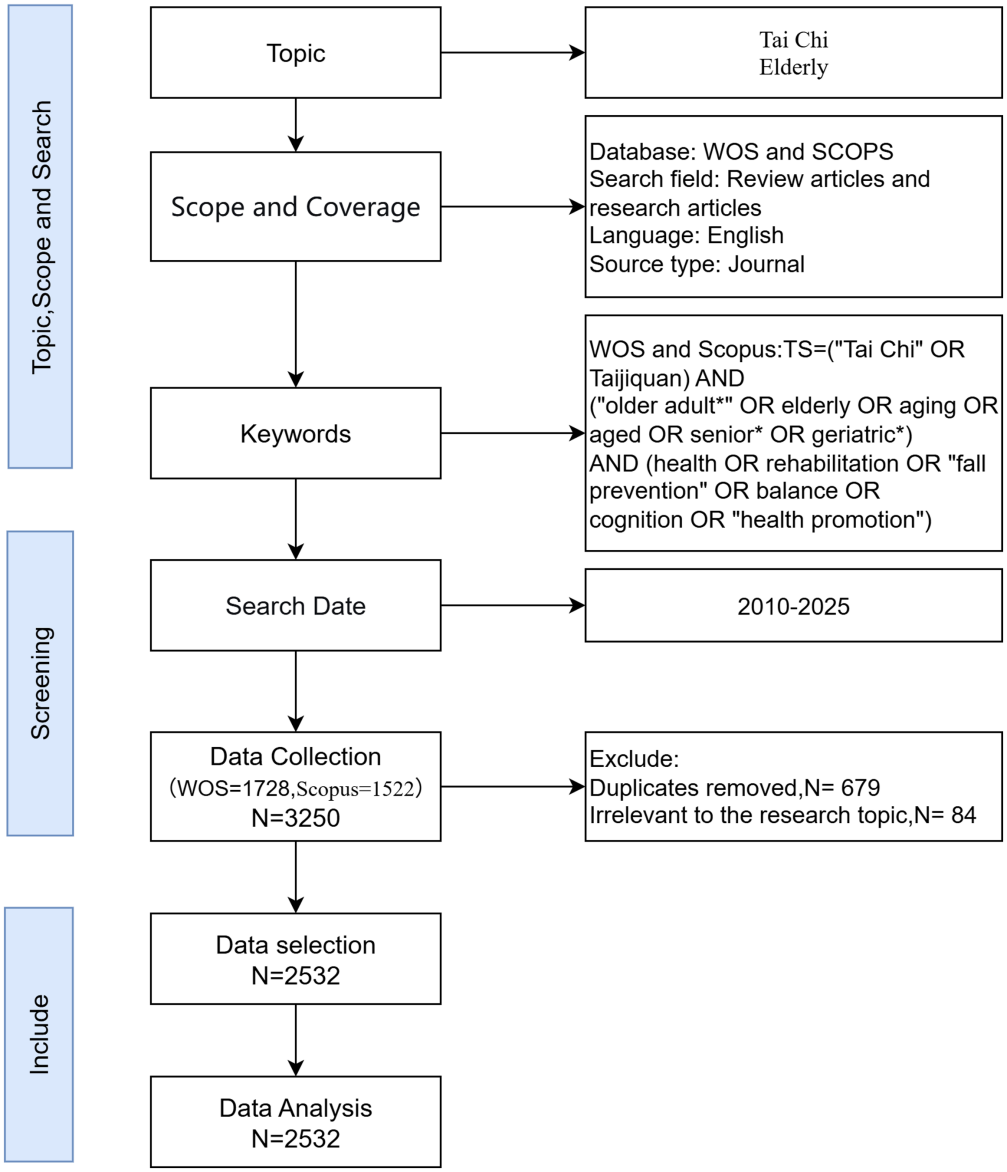
Flow diagram of the search strategy.

This study used a bibliometric analysis method, retrieving literature on the role of Tai Chi in promoting the health of older adults from two authoritative international databases: Web of Science (WoS) and Scopus. These databases house an extensive collection of high-quality research papers and are highly compatible with various bibliometric software, making them particularly suitable for citation analysis. We conducted a systematic literature search of the WoS Core Collection and the Scopus database, adhering to the PRISMA (Preferred Reporting Items for Systematic reviews and Meta-Analyses) guidelines. The following keyword combinations were used: TITLE-ABS-KEY (Tai Chi OR Taijiquan) AND TITLE-ABS-KEY (older adult OR older adult(s) OR aging OR aged OR senior OR geriatric) AND TITLE-ABS-KEY (health OR rehabilitation OR fall prevention OR balance OR cognition OR health promotion). To ensure the timeliness of information in the research field, the literature search and data extraction were conducted on December 30, 2025. The search period covers articles published between January 1, 2010, and December 30, 2025. Due to indexing delays, the bibliographic data for 2025 may be incomplete. Inclusion criteria: (1) article or reviews written in the English language; (2) involving research subjects aged 60 years or older; (3) using Tai Chi (regardless of school) as the intervention content; (4) outcomes included health-related indicators such as physical health, mental health, cognitive function, balance ability, chronic diseases, or rehabilitation. Exclusion criteria: (1) duplicate literature; (2) unrelated topics; (3) literature types other than a research paper or review article. Applying these criteria, a total of 3,250 articles were initially retrieved from the WoS and Scopus databases (WoS: 1723; Scopus: 1522). To ensure the integrity and accuracy of the data analysis, a rigorous data-cleansing process was implemented. Due to the records from WOS and Scopus were exported in Plain Text and RIS formats, respectively. First, duplicate records between the WoS and Scopus databases were identified and removed using the ‘Remove Duplicates’ function in CiteSpace (version 6.4. R1), followed by manual verification of titles, authors, and DOIs. Second, institutional name standardization was performed to merge synonymous entries and address variations in naming conventions. For instance, different variants (e.g., ‘Shanghai Univ Sport’ and ‘Shanghai University of Sport’) were unified, and subordinate affiliations (e.g., ‘Harvard Medical School’) were merged into their parent institution (‘Harvard University’). After literature deduplication, cleaning, and screening to remove additional ineligible literature according to the inclusion and exclusion criteria, 2,532 eligible articles were included in the analysis. Figure 1 shows the flowchart of the retrieval strategy analysis.

### 2.2 Data analysis

We used VOSviewer (version 1.6.20) to conduct a systematic bibliometric analysis of the included literature, constructing a collaborative network of authors, institutions, and countries, as well as a keyword co-occurrence network. High-impact authors and journals were identified through cocitation and literature coupling analysis to explore the research hotspots and development trends in this field. Network, overlay, and density views were used to visualize research topics, collaboration patterns, and hotspot evolution. To ensure the robustness and interpretability of the results, the analysis parameters followed VOSviewer’s recommended settings, including the minimum number of documents, minimum co-occurrence frequency, and standardized edge weights. Emerging word detection was performed using CiteSpace’s Kleinberg Burst Detection algorithm to identify keywords that experienced a significant increase in frequency during specific periods, thereby revealing emerging topics and research frontiers within the field.

## 3 Results

### 3.1 Annual publication trends

**Figure 2:**
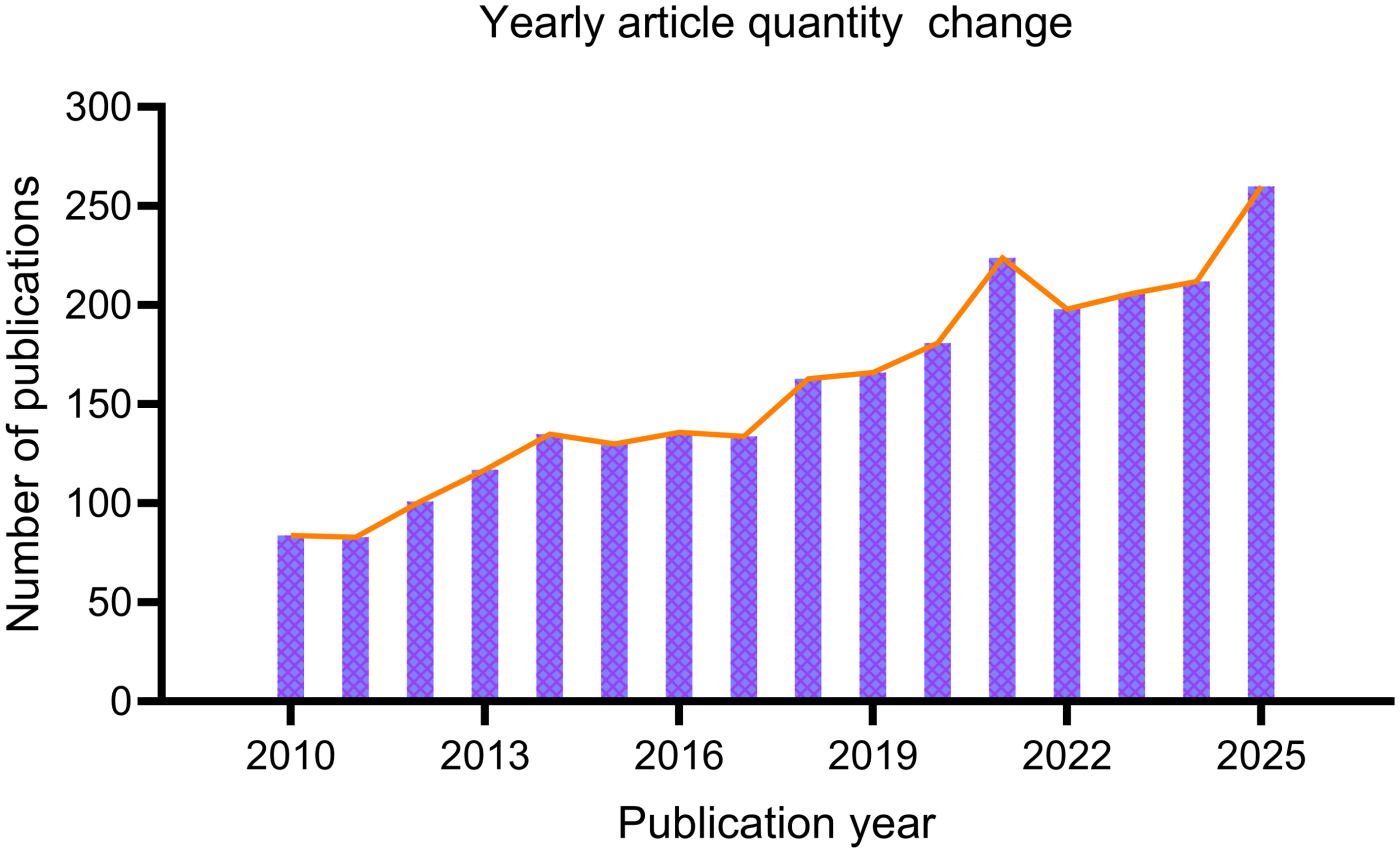
Annual publication trend of research on Tai Chi and the health of the older adult(s) (2010–2025).

Figure 2 illustrates the annual publication volume of research on the older adult(s) and Tai Chi, which exhibited an overall upward trend from 2010 to 2025, with steady growth across various stages of development. The annual publication volume gradually increased from approximately 80 articles to 120 articles between 2010 and 2014, indicating that the field was in its initial exploratory stage. From 2015 to 2018, the publication volume fluctuated slightly between 130 and 150 articles per year, but still showed an overall upward trend, reflecting a structural expansion stage. Publication volume grew significantly between 2019 and 2021, rising from approximately 160 to over 200 articles, reflecting the field’s rapid expansion. While 2022 saw a slight decline, the publication volume remained high. Between 2023 and 2025, the number of related papers rebounded rapidly, reaching a peak in 2025 (approximately 260–270 papers), indicating that the field has entered an accelerated phase of development. This sustained growth trend highlights the increasing academic attention to the use of Tai Chi interventions in the health of older adults.

### 3.2 Country and institutional analysis

**Figure 3:**
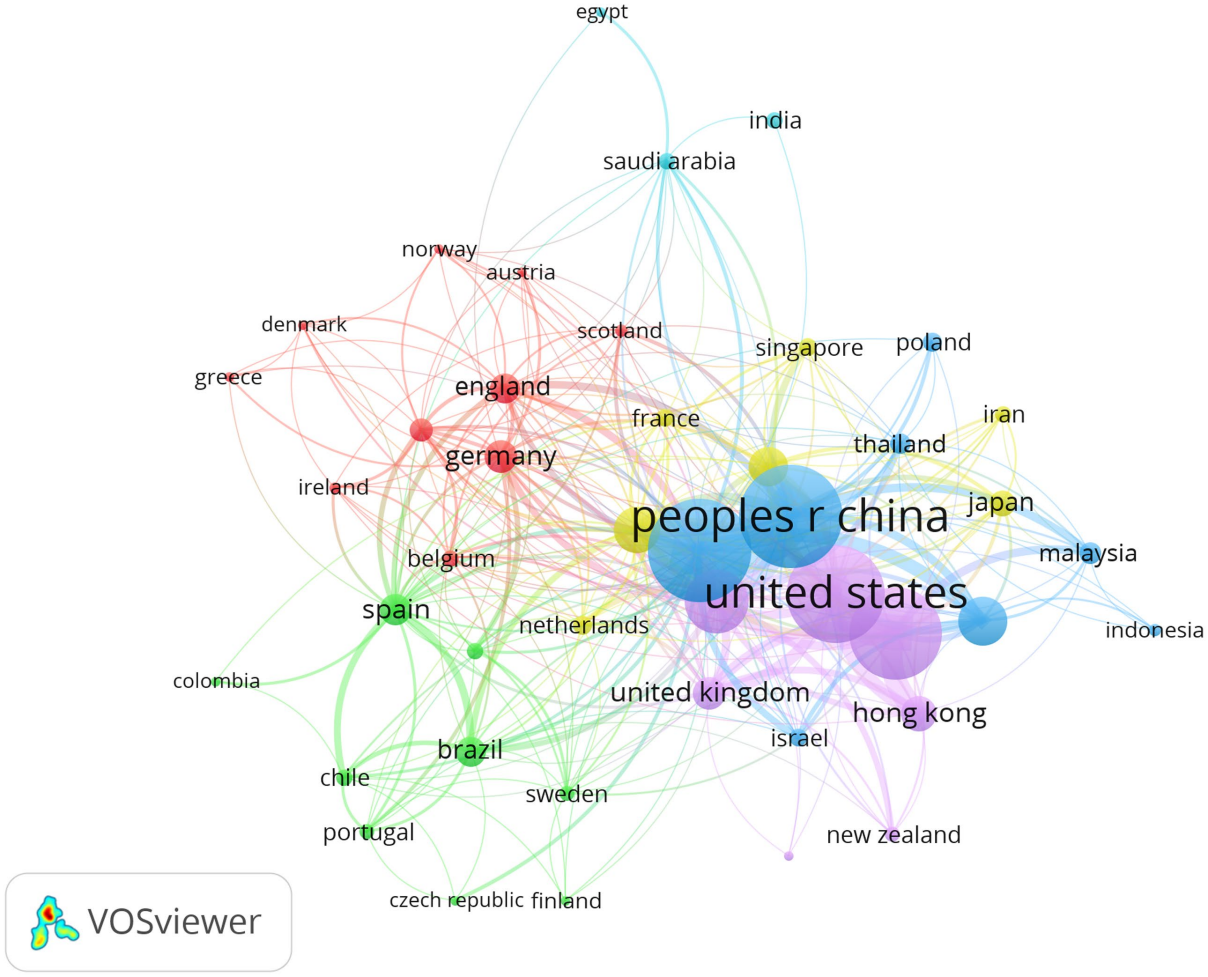
Network view of national document issuance cooperation.

Table 1 lists the top 10 countries in terms of the number of papers published among the 121 contributing countries. China topped the list with 1,034 papers (41%), followed by the United States with 865 (34%), Australia with 207 (8%), the United Kingdom with 125 (5%), and Canada with 119 (5%). The national-level cooperation network (Figure 3) shows that China and the United States occupy a central position, forming a dense pattern of international cooperation. Findings in Europe and Australia are also primarily characterized by transnational collaborations. This network structure suggests a dual-core model: China provides a strong foundation of traditional knowledge and practices, while the United States drives clinical and public health-oriented research.

**Table 1:** Distribution of publications by country.

Rank	Country	Publications	Percentage (%)
1	China	1,034	41%
2	USA	865	34%
3	Australia	207	8%
4	United Kingdom	125	5%
5	Canada	119	5%
6	South Korea	93	4%
7	Germany	66	3%
8	Spain	65	3%
9	Brazil	59	2%
10	Japan	46	2%

Percentage is calculated based on the total number of publications (*n* = 2,532).

**Figure 4:**
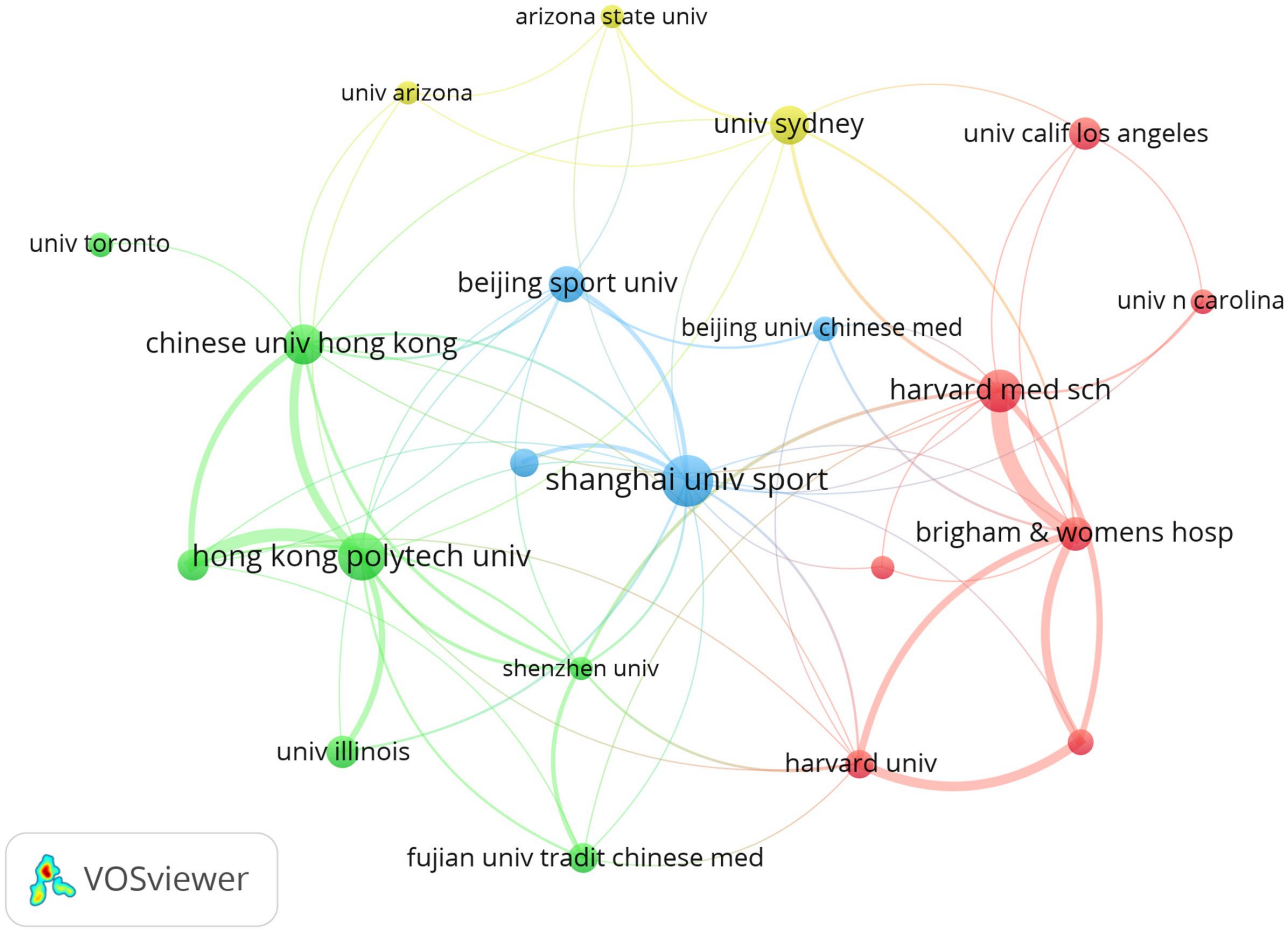
Network diagram of institutional publication volume and cooperation relationships.

The institutional collaboration analysis presented in Table 2 and Figure 4 shows a predominant concentration of high-productivity institutions in China, including Shanghai University of Sport, Beijing Sport University, and universities in Hong Kong, as well as in the United States, particularly within the Harvard University system. These institutions form unique collaborative clusters, highlighting regional differences and the existence of core institutional centers for global research on Tai Chi among older adults.

**Table 2:** Top 10 productive institutions in Tai Chi research on older adults (2010–2025).

Rank	Institution	Docs (n)	Cites (n)	TLS
1	Shanghai University of Sport	52	969	26
2	The Hong Kong Polytechnic University	46	286	39
3	Harvard Medical School	38	230	39
4	The Chinese University of Hong Kong	35	612	27
5	The University of Sydney	32	444	12
6	Beijing Sport University	29	402	13
7	Brigham and Women’s Hospital	26	791	42
8	Department of Medicine	24	624	0
9	University of California, Los Angeles	24	584	4
10	University of Illinois	24	873	9

Docs = number of publications; Cites = total citations; TLS = total link strength based on journal co-citation analysis.

### 3.3 Author and journal analysis

#### 3.3.1 Author analysis

Table 3 lists the top 10 most prolific authors among the 2,532 included articles. Peter M. Wayne (31 articles) has the most publications, followed by Helen Lavretsky and Li Fuzhong with 25 articles each, and Jing Tao, Chenchen Wang, and Liye Zou with 22 articles each. These authors are key contributors to research on Tai Chi in older adults.

**Table 3:** Top productive authors in Tai Chi research on older adults (2010–2025).

Rank	Author	Docs (n)	Cites (n)	TLS
1	Wayne, Peter M.	31	446	53
2	Lavretsky, Helen	25	126	28
3	Li, Fuzhong	25	813	27
4	Tao, Jing	22	255	68
5	Wang, Chenchen	22	597	39
6	Zou, Liye	22	207	6
7	Chen, Lidian	19	861	62
8	Tsang, William W. N.	19	477	18
9	Li, Li	18	291	23
10	Yeh, Gloria Y.	18	264	17

Docs = number of publications; Cites = total citations; TLS = total link strength based on journal co-citation analysis.

**Figure 5:**
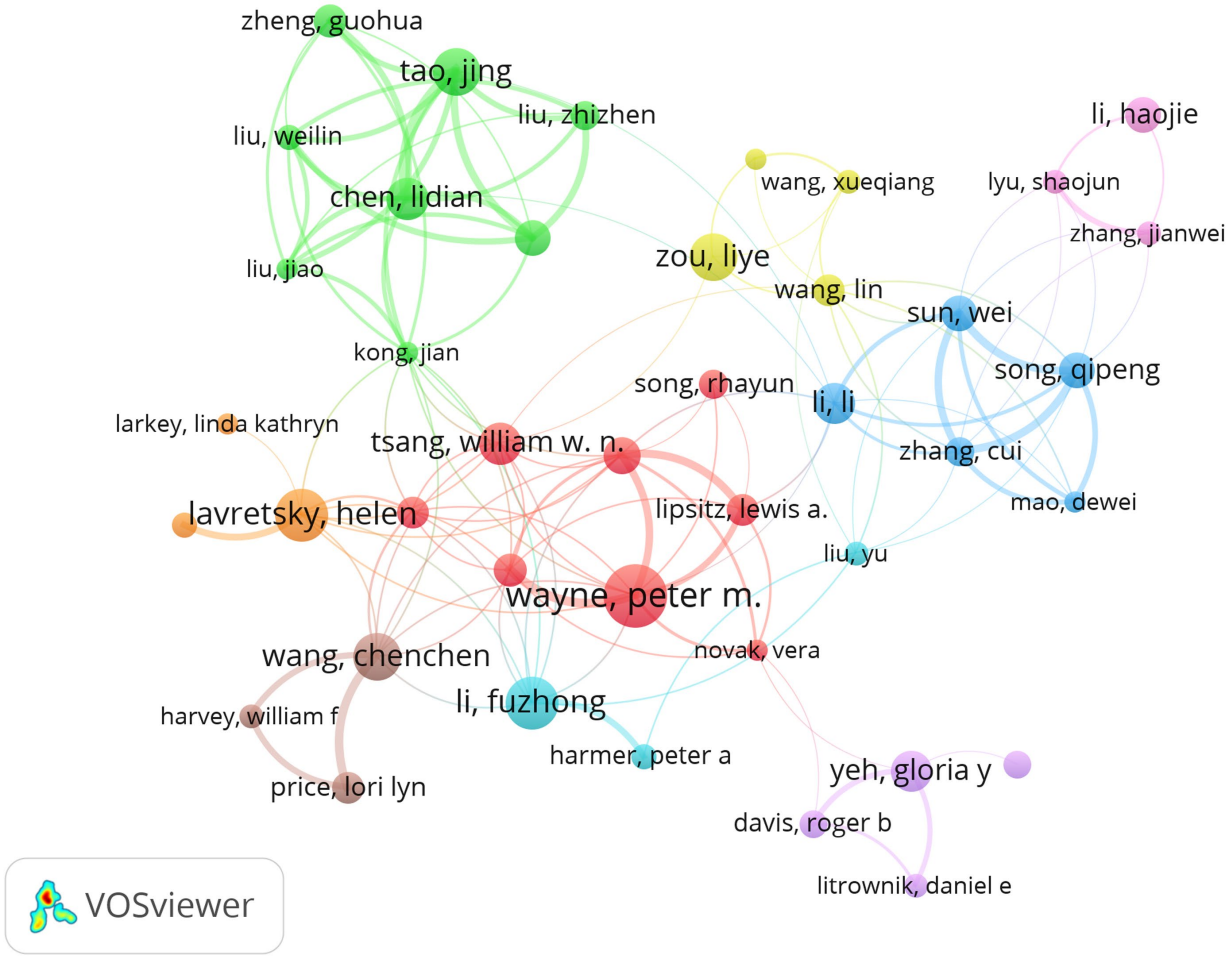
Network diagram of author publication volume and cooperation relationship.

Figure 5 reveals three primary research clusters within the author collaboration network. The first cluster, represented by Wayne, Wang, and Yeh, focuses on clinical and integrative medicine, with a particular emphasis on randomized controlled trials (RCTs) of Tai Chi interventions. The second cluster, represented by Lavretsky and Li Fuzhong, investigates the effects of Tai Chi interventions on age-related outcomes and mental health, including depression and mental well-being in older adults. The third cluster, represented by Jing Tao, Liye Zou, and Lidian Chen, focuses on the effects of Tai Chi on rehabilitation, traditional Chinese medicine, and exercise interventions in older adults. This author collaboration network demonstrates both close cooperation and the variety of topics between China and the United States. Furthermore, the significant interdisciplinary integration reflects the diversity of Tai Chi research on the older adult(s), spanning clinical, psychological, and rehabilitation fields.

#### 3.3.2 Journal analysis

**Figure 6:**
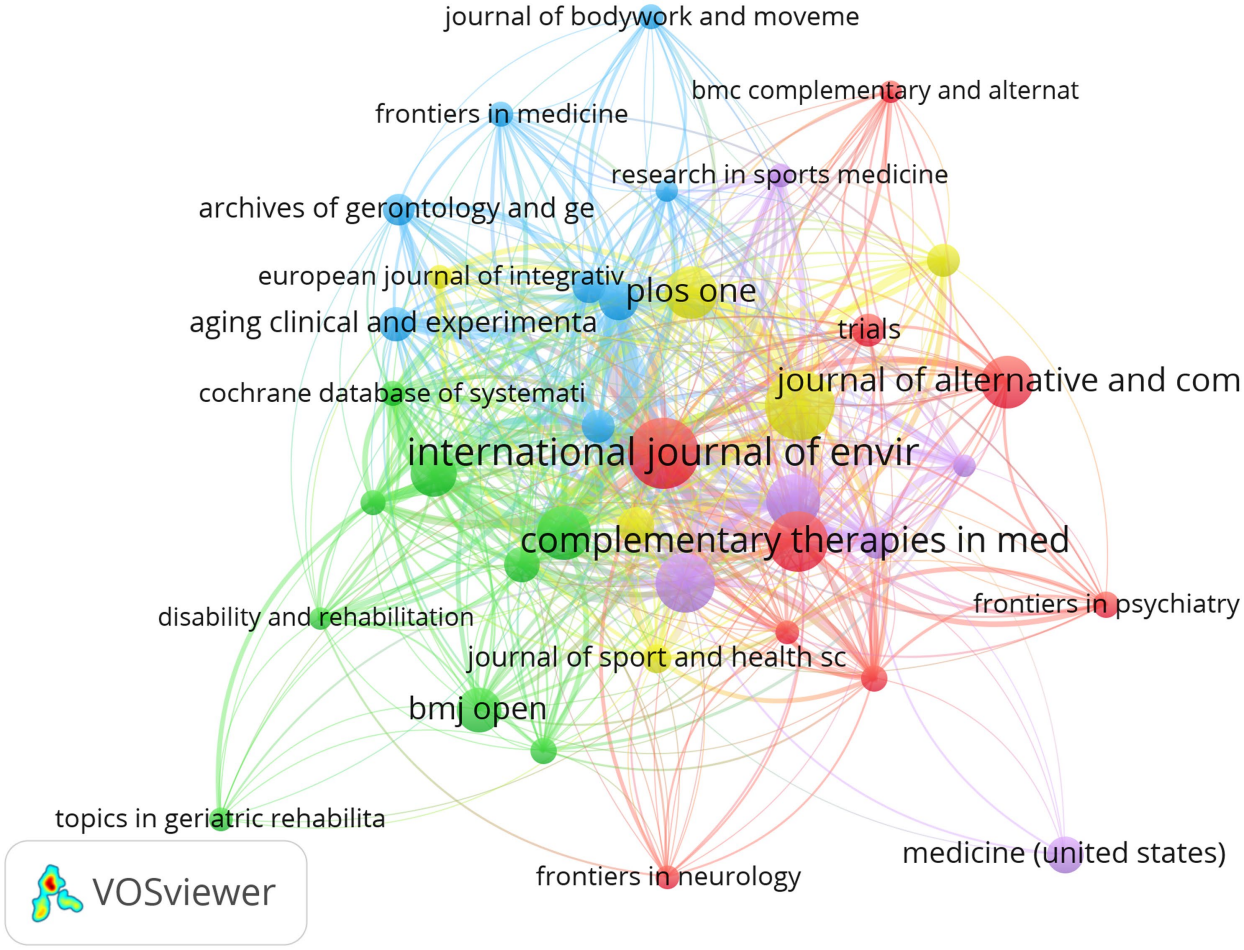
Network diagram of cooperation relationships in journal publication volume.

Table 4 summarizes the leading journals publishing research on Tai Chi in the context of health promotion among older adults. The *International Journal of Environmental Research and Public Health* published the largest number of articles in this field (55 papers; total link strength (TLS) = 8,195), underscoring its central role in disseminating Tai Chi–related public health research. *Evidence-Based Complementary and Alternative Medicine* ranked second (54 papers; TLS = 7,432), representing a major outlet for complementary and alternative medicine studies. *Complementary Therapies in Medicine* (45 papers) and *Frontiers in Public Health* (44 papers) also made substantial contributions, exhibiting strong citation performance and cocitation linkages. Aging-related journals, including *Journal of Aging and Physical Activity* (1,376 citations) and *Frontiers in Aging Neuroscience* (1,478 citations), demonstrated the highest citation impact, highlighting the relevance of Tai Chi research within gerontology and neuroscience. In addition, multidisciplinary journals such as *PLOS ONE*, *BMC Geriatrics*, and *BMJ Open* showed relatively high citation counts and link strengths, indicating broad dissemination across public health, geriatrics, neuroscience, and complementary medicine (Figure 6). Overall, the journal distribution reflects the pronounced interdisciplinary nature of Tai Chi research in older adult health.

**Table 4:** Top journals publishing Tai Chi research (2010–2025).

Rank	Journal	Docs (n)	Cites (n)	TLS	IF	*Q*
1	International Journal of Environmental Research and Public Health	55	246	8,195	Dropped*	
2	Evidence-Based Complementary and Alternative Medicine	54	231	7,432	Dropped*	
3	Complementary Therapies in Medicine	45	491	3,458	3.5	1
4	Frontiers in Public Health	44	182	5,861	3.4	1
5	Journal of Aging and Physical Activity	40	1,376	6,771	1.5	2
6	Frontiers in Aging Neuroscience	39	1,478	4,175	4.5	1
7	Journal of Integrative and Complementary Medicine (formerly JACM)	38	934	626	1.7	2
8	PLOS ONE	38	192	3,951	2.6	1
9	BMC Geriatrics	33	798	5,218	3.8	2
10	BMJ Open	31	401	1,574	2.3	2

JACM = Journal of Alternative and Complementary Medicine; Docs = number of publications; Cites = total citations; TLS = total link strength based on journal co-citation analysis; IF = Impact Factor; Q = Quartile, Quartile are based on the latest Journal Citation Reports (JCR 2025). Dropped* = International Journal of Environmental Research and Public Health and Evidence-Based Complementary and Alternative Medicine was delisted from Science Citation Index in 2023. It is included here to reflect its historical contribution to the total publication volume during the early-to-mid study period.

### 3.4 Keyword co-occurrence and research hotspots

**Figure 7:**
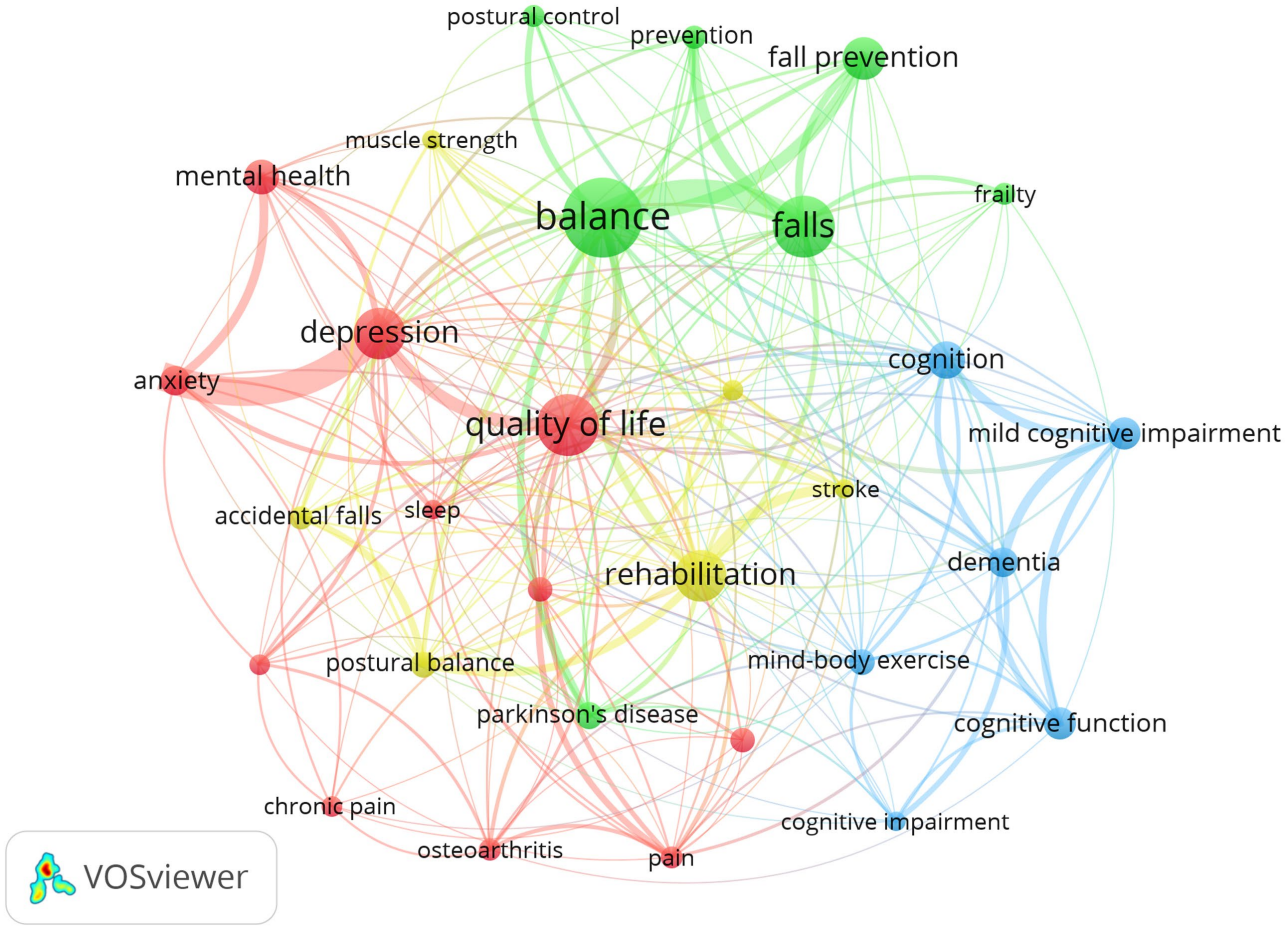
Keyword co-occurrence and research hotspot clustering diagram.

Table 5 lists the 10 most frequently occurring keywords with the highest overall link strength. These include “balance,” “quality of life,” “falls,” “depression,” and “rehabilitation.” Figure 7 depicts the keyword clustering network diagram, illustrating how these core keywords form four interconnected main research clusters. Cluster 1 focuses on balance function and fall prevention, with balance, fall, and fall prevention serving as central nodes. This clustering indicates strong evidence supporting the role of Tai Chi in improving postural control and reducing fall risk in older adults. Cluster 2 centers on cognitive outcomes, including cognition, mild cognitive impairment, and dementia, highlighting the potential role of Tai Chi as a mind–body exercise in alleviating cognitive decline and neurodegenerative diseases. Cluster 3 emphasizes mental health through keywords such as depression, anxiety, and mental health, suggesting that Tai Chi is beneficial for mood regulation and overall health. Cluster 4 keywords relate to rehabilitation and the management of chronic diseases, including stroke and osteoporosis, reflecting the application of Tai Chi in clinical rehabilitation and long-term health management. The keyword co-occurrence pattern indicates that research on Tai Chi in older adults has developed into a comprehensive, multidimensional framework for health promotion.

**Table 5:** Most frequent keywords in Tai Chi research on older adults (2010–2025).

Rank	Keyword	Occurrences	TLS
1	Balance	175	120
2	Quality of life	127	82
3	Falls	127	89
4	Depression	103	103
5	Rehabilitation	101	88
6	Fall prevention	81	41
7	Cognition	68	56
8	Mental health	64	33
9	Cognitive function	57	34
10	Mild cognitive impairment	56	48

Occurrences, frequency of keyword appearance; TLS, total link strength indicating the degree of co-occurrence connections in VOSviewer.

### 3.5 Topic evolution and keyword explosion analysis

**Figure 8:**
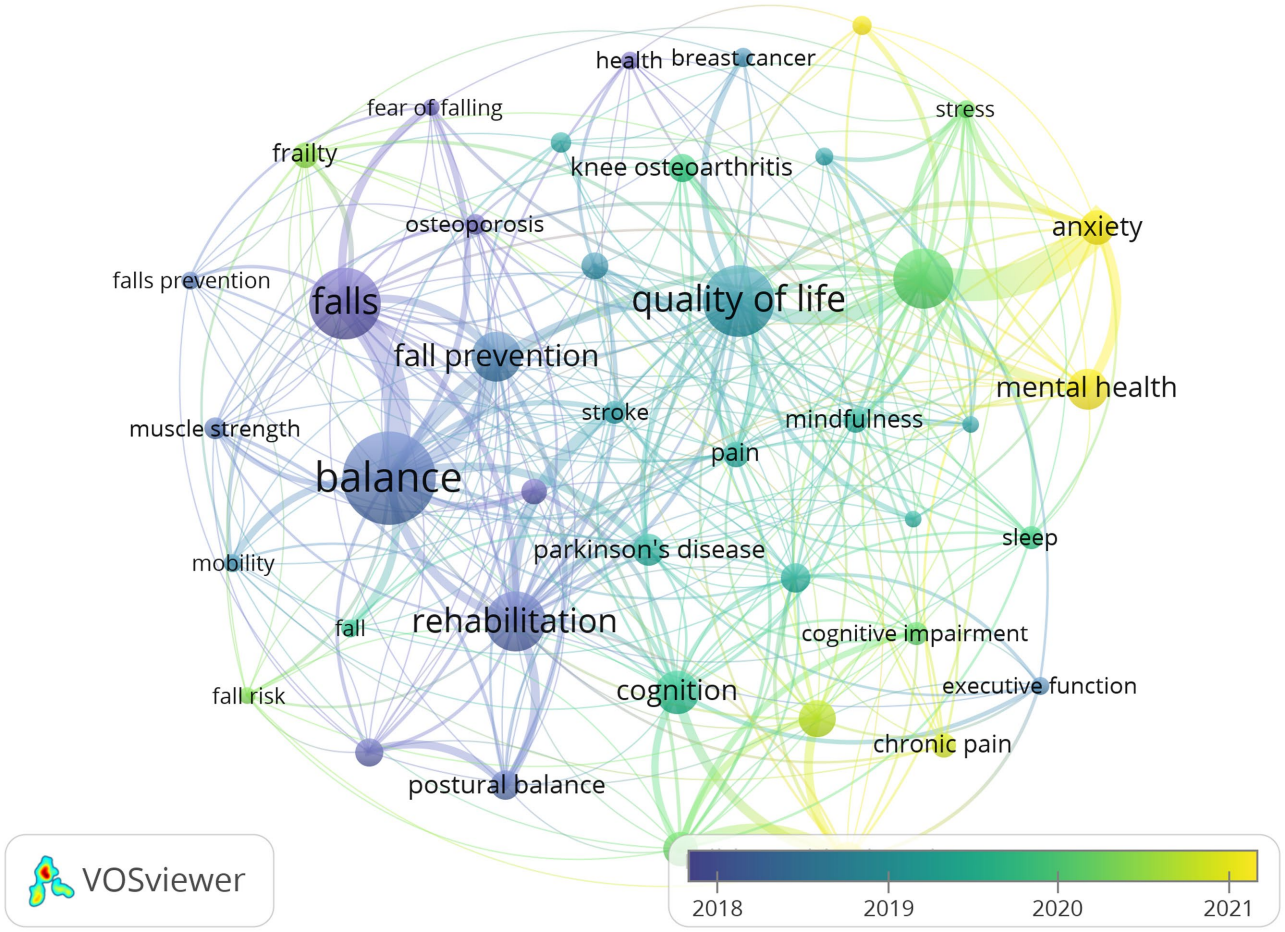
Overlay visualization of keyword co-occurrence in Tai Chi research on the older adult(s).

**Figure 9:**
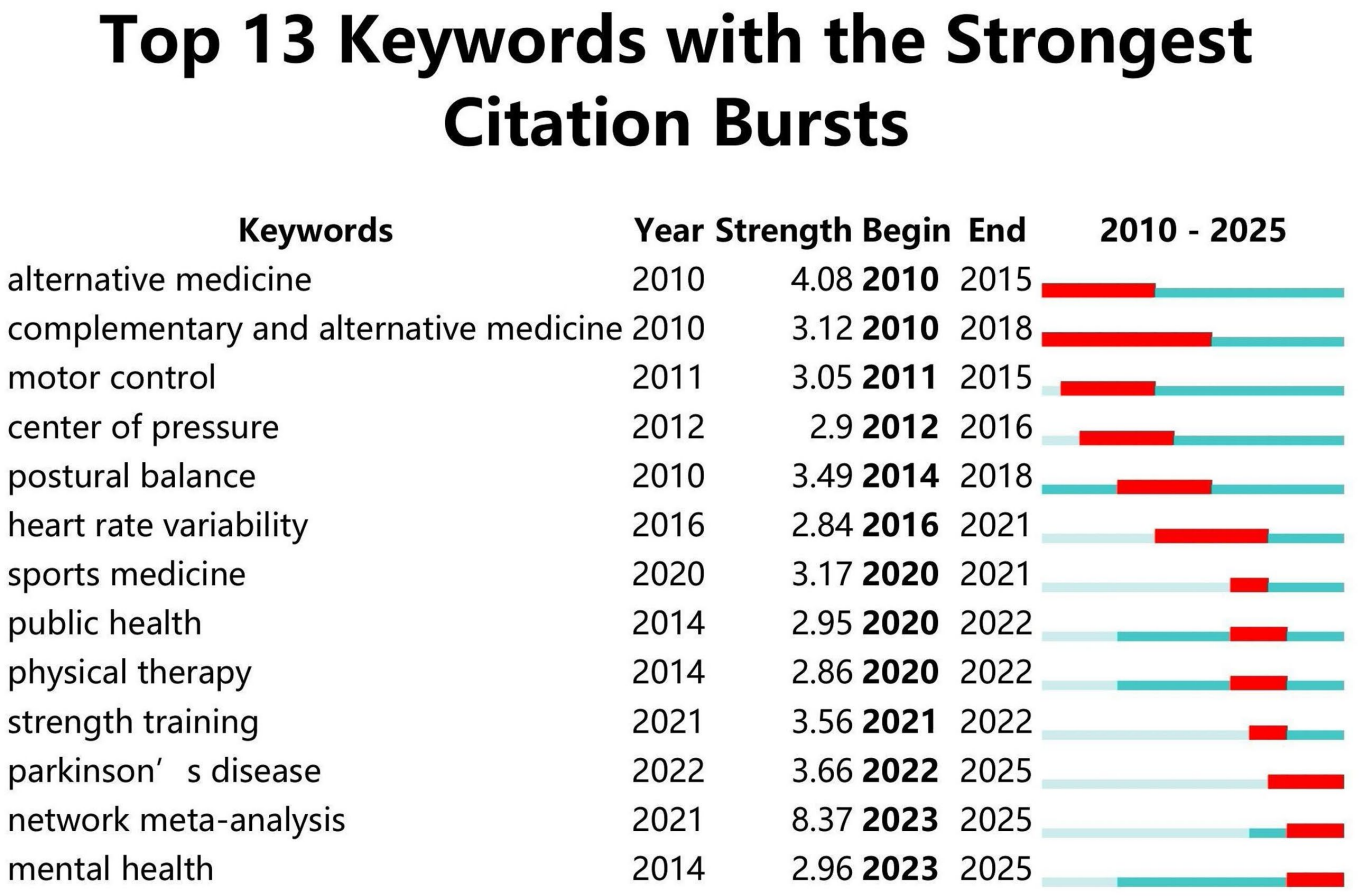
Keywords with the highest intensity emerging in the study of Tai Chi in the older adult(s).

Figure 8 illustrates the keyword overlay visualization network. Although the software analysis spans a limited timeframe, it clearly reveals the temporal evolution of research on Tai Chi in older adults. Early research (around 2018–2019) focused primarily on physical function, including balance, falls, fall prevention, and rehabilitation. These research topics reflected the then-current emphasis on Tai Chi as an intervention to reduce fall risk and improve mobility in older adults. Keywords related to interventions in older adults, such as quality of life, cognition, mental health, sleep, and mindfulness, received widespread attention, indicating that Tai Chi research has shifted from focusing on single physical outcomes to promoting overall health. The keyword explosion analysis shown in Figure 9 further reveals emerging research frontiers. Early-emerging keywords were mainly related to alternative medicine and postural balance, while recently emerging keywords focus on network meta-analysis, mental health, Parkinson’s disease, and strength training. These newer terms indicate that Tai Chi research is gradually moving toward an evidence-based approach and is increasingly focused on the neurological, psychological, and comprehensive rehabilitation effects of Tai Chi on older adults.

## 4 Discussion

### 4.1 Development of Tai Chi research in the context of global healthy aging

The bibliometric analysis showed that research on Tai Chi’s health benefits for older adults exhibited sustained growth between 2010 and 2025, indicating increasing scholarly attention to healthy aging within the global public health field. Prior to 2015, the gradual increase in publications highlighted a primary focus on the feasibility and safety of Tai Chi interventions. Consequently, a substantial body of literature concentrated on fall prevention and balance improvement (24–26), as these outcomes represent the most direct and quantifiable health indicators in older populations. The marked increase in publications after 2019 coincided with intensified global concern regarding population aging (27). Scholars increasingly focus on non-pharmacological interventions to reduce the medical burden of chronic diseases and disabilities in the older adult(s), such as the fitness benefits provided by improved quality of life (28), cognitive function (29), mental health (30), sleep (20, 31), and mindfulness (32). Notably, Tai Chi is inexpensive to practice, requires minimal space and equipment, and has no specific target audience requirements (33), making it particularly suitable for community-based and preventive health models (34). Therefore, the surge in related publications may reflect the transition of Tai Chi from an auxiliary form of exercise to a prominent focus within global strategies for healthy aging. While the surge in publications underscores a robust scholarly interest in health resource optimization, the field must now pivot from mere efficacy validation toward the translation of these findings into the complex socio-economic realities of aging populations.

### 4.2 Dual-core national structure and the logic of international organization cooperation

The bibliometric results show that China (41%) and the United States (34%) lead in the number of papers published and the strength of their cooperation networks (Table 1); both play a pivotal role in international cooperation networks (Figure 3). China’s primary focus is on modernizing Tai Chi based on traditional knowledge, with institutions such as Shanghai University of Sport, Beijing Sport University, and the University of Hong Kong representing this approach (Table 2). Empirical studies by Chinese scholars on the beneficial effects of Tai Chi on muscle strength (35, 36), proprioception (37), and gait stability in older adults (38, 39) have contributed extensive data regarding its application as a potential exercise prescription for global health promotion. Meanwhile, the United States, centered around Harvard Medical School, has spearheaded the clinical integration and mechanistic deepening of the field. The country’s high citation counts and TLS reflect a dominant academic discourse, particularly through rigorous RCTs. These studies have significantly advanced the discussion on the potential mechanism by which Tai Chi influences neural plasticity (40, 41), immune regulation (42, 43), and inflammatory cytokines in older adults (44). Such mechanism-driven research has significantly elevated the empirical standing of Tai Chi within modern rehabilitation medicine. The bilateral collaboration between China and the US has thus forged a dual-core system. The dense collaborative chains illustrated in Figures 3, 4 reflect a logical consensus within the international academic community regarding the global healthcare burden of aging. The growing participation of countries such as Australia, the United Kingdom, and Canada further demonstrates the recognition of Tai Chi as a low-cost, non-pharmacological, widely accessible, and cost-effective health promotion intervention (44). This pattern presages a strategic shift in Tai Chi research, transitioning from a regional practice into a globally applicable health intervention strategy for public health promotion. Despite the dominance of the dual-core structure, the geographical imbalance in publication volume risks marginalizing vulnerable nations. To ensure public health equity, efforts must be made to secure cultural and economic support for Tai Chi in regions where resources are most scarce and population aging is most rapid.

### 4.3 Core leading forces and interdisciplinary knowledge transfer

The bibliometric analysis demonstrates the evolution of Tai Chi research into a mature field, driven by leading scholars, with multidisciplinary support. The research paradigm is advancing from a single rehabilitation method to a deeper integration of multidimensional mechanisms and practices. With Peter M. Wayne, Li Fuzhong, and Tao Jing as central figures, a robust empirical foundation for the global collaborative network in this field has been established (Table 3). Researchers such as Wayne and Wang advanced the evidence base for the physical and mental health benefits of Tai Chi through RCTs (45, 46), while Li et al. (47) demonstrated through their highly cited works the critical role of Tai Chi in enhancing postural stability and preventing falls among older adults through community-based intervention studies, informing public health policy development. With the growing research interest in the health-promoting effects of Tai Chi, empirical studies have increasingly shifted from functional outcome evaluation toward in-depth exploration of positive neurobiological mechanisms. As Table 4 shows, the high citation frequency of journals such as *Frontiers in Aging Neuroscience* (Cites > 1,300) indicates that the academic community has begun to focus on Tai Chi’s effects on neuroplasticity. The prevailing academic consensus, as reflected in high-impact publications, suggests that Tai Chi is associated with favorable structural and functional changes in the aging brain (48). This conceptual transition—from physical exercise to neural network optimization—provides a solid biological foundation for Tai Chi–based interventions that target mild cognitive impairment (MCI) (49). As research perspectives become more socially embedded, the prominence of public health journals such as the *International Journal of Environmental Research and Public Health* (*IJERPH*) underscores the growing role of Tai Chi in global health strategies. The low cost, non-pharmacological nature, and high accessibility of Tai Chi mean it has substantial potential to alleviate the healthcare burden associated with aging-related chronic diseases worldwide (50). This paradigm shift—from an adjunctive therapy to a population-level health strategy—has not only stimulated an expansion of empirical research but has also contributed traditional Chinese sports wisdom to the global agenda on healthy aging. While current research paradigms are academically mature, these findings have failed to fully consider the ecological effects of Tai Chi as a lifelong habit. Therefore, translating neuroplasticity research findings into scalable public health solutions remains an ongoing challenge, requiring a shift from purely clinical observation to practical science.

### 4.4 Evolution of research hotspots and multidimensional health promotion models

Keyword co-occurrence analysis (Table 5) and clustering networks (Figure 7) indicate that Tai Chi research has progressively shifted from a focus on isolated functional benefits toward an in-depth exploration of integrated physiological psychological cognitive and social effects. The exceptionally high co-occurrence frequency of “balance” and “falls” in Cluster 1 (Table 5) highlights that Tai Chi remains a core thematic cluster and a frequently validated intervention for reducing fall risk among older adults. Prior research has demonstrated that Tai Chi interventions significantly reduce fall incidence by 24–34% (36) and outperform conventional stretching or resistance training in improving postural control (47). Cluster 4 extends the application of Tai Chi into clinical domains including stroke osteoporosis and cardiovascular diseases reflecting its evolution from a general exercise modality into a targeted intervention for specific pathological conditions. Earlier research found that Tai Chi is an effective rehabilitative strategy for patients with coronary heart disease (51, 52). The prominence of Clusters 2 and 3 further suggests a paradigm shift from broad investigations of basic physical function toward deeper mind–body integration. At the cognitive level Tai Chi—characterized by sequential movement memory and spatial orientation—is extensively documented in the literature to correlate with improvements in individuals with MCI and to induce neuroplastic changes in key brain regions such as the hippocampus and prefrontal cortex (45). Recent bibliometric evidence indicates that the exploration of the neurobiological mechanisms underlying mind–body exercises has emerged as a prominent research trend. Interventions such as Tai Chi are increasingly recognized for their capacity to modulate neural excitability and structural connectivity, which are essential for delaying the progression of MCI (53). Concurrently research has shown that Tai Chi exhibits significant effects in alleviating depression and anxiety potentially by reducing cortisol levels and proinflammatory cytokines (e.g., IL-6) thereby elucidating the mechanisms by which physical activity couples with neuroendocrine regulation. These effects collectively constitute a central pathway for improving overall quality of life in older adults (46, 54). This multidimensional evidence structure signals the emergence of a new paradigm for addressing global aging challenges directly aligning with the WHO’s strategic emphasis on maintaining functional independence in older populations and offering an efficient low-cost intervention to mitigate the societal and healthcare burdens associated with population aging (55, 56).

### 4.5 Thematic evolution pathways and emerging research frontiers

Overlay visualization of keywords (Figure 8) and burst-term analysis (Figure 9) reveal a progressive trajectory in research on Tai Chi and healthy aging, moving from foundational functional validation to multidimensional health promotion and, ultimately, toward precision-oriented, evidence-based practice. Prior to 2019, research was highly concentrated on terms such as “balance,” “falls,” and “rehabilitation,” reflecting a strong scholarly focus on the role of Tai Chi in reducing fall risk and improving physical function among older adults (57). After 2020, the emergence and rapid growth of keywords such as “quality of life,” “cognition,” and “mindfulness” are indicative of the growing evidence that Tai Chi interventions also improve sleep quality, mindfulness levels, and psychological resilience. This shift reflects increasing academic attention to the multidimensional components of successful aging within aging societies (58), signaling a transition from single-outcome physical interventions toward comprehensive health benefits. The evolution of burst keywords further highlights the expansion of research frontiers into complex disease management and precision medicine. As shown in Figure 9, burst terms shifted from early emphases on alternative medicine to more disease- and method-specific terms such as “Parkinson’s disease,” “strength training,” and “network meta-analysis.” The sustained prominence of neurodegenerative conditions in the literature, particularly Parkinson’s disease, suggests that Tai Chi is becoming increasingly integrated into mainstream neurorehabilitation research. The research trend suggests a growing focus on Tai Chi’s potential to induce neural compensatory mechanisms, improving gait stability and reducing bradykinesia in affected populations (47). The emergence of network meta-analysis reflects methodological maturation, indicating a move beyond isolated efficacy assessments toward comparative evaluations of intervention intensity, frequency, and modality. This development provides a more solid scientific foundation for optimizing Tai Chi–based exercise prescriptions in clinical and public health fields. With the widespread application of digital and artificial intelligence technologies in recent years, research suggests that the transmission, teaching, and assessment of Tai Chi can be achieved through virtual reality (VR), augmented reality (AR), motion capture (59, 60), and AI-corrected movements (61). Simultaneously, the combination of VR, wearable devices, and telemedicine has opened new avenues for applying Tai Chi in digital health promotion (62). While the digital frontier brings innovation, it can also create a “digital divide,” excluding the most vulnerable older adult(s) populations. Furthermore, over-reliance on complex methods such as network meta-analysis should not overlook the high heterogeneity of different Tai Chi styles. Future research must strike a balance between technological precision and inclusive participation to ensure that “precision medicine” benefits everyone.

### 4.6 Limitations and future study

This study has several limitations. First, the search was limited to English-language literature in the WoS and Scopus databases, potentially overlooking significant non-English studies, particularly those from China. Furthermore, considering the inherent time lag in database indexing, the 2025 search results represent most records and may not reflect the full output for the entire year. Second, the analytical tools prioritize recent trends, offering limited insight into long-term thematic evolution. Notably, while several high-output journals in this field were recently excluded from the Science Citation Index database, the migration of research to mainstream high-impact journals, such as *Frontiers in Aging Neuroscience*, signifies a progressive enhancement in evidentiary quality. Future research should prioritize mechanism-driven and rigorously designed RCTs to improve reproducibility and the overall grade of evidence.

## 5 Conclusion

This study systematically maps the global research landscape on the role of Tai Chi in older adult(s) health from 2010 to 2025. The results show that the number of research findings on the health promotion of Tai Chi for the older adult(s) has been accelerating over time, forming a collaborative network centered on China and the United States. The field has shifted from solely verifying physical function to exploring multidimensional holistic health promotion that encompasses neurological, cognitive, and psychological improvements, forming a dual-driven research pattern with China and the United States at its core. Research frontiers point toward the deepening of personalized exercise prescriptions for specific precision rehabilitation and digital rehabilitation methods. While the quality of some high-output journals has fluctuated, the mainstreaming of research directions in recent years signifies an overall improvement in the level of evidence. The research results support the inclusion of Tai Chi as a low-cost, high-efficiency public health intervention in global public health strategies to address the social challenges posed by population aging.

## Data Availability

The original contributions presented in the study are included in the article/Supplementary material, further inquiries can be directed to the corresponding authors.
