# Supplementary material for: Global research trends and hotspots in the health effects of Tai Chi on older adults: a bibliometric analysis (2010–2025)
Source: Front Public Health. 2026 Mar 24;14:1799937. doi: 10.3389/fpubh.2026.1799937 (PMC13053515; doi:10.3389/fpubh.2026.1799937)
Supplement: Supplementary file 1 [file Data_Sheet_1.PDF]

Supplementary Material

Global Research Trends and Hotspots in the Health Effects of Tai Chi on Older Adults: A Bibliometric Analysis (2010–2025)

Supplementary Data

1 Search Strategy

Data Source: Web of Science (WoS) Core Collection and Scopus

Time Span: 2010-01-01 to 2025-12-30

Retrieval Date: 2025-12-30

2 Web of Science (WoS) Core Collection

Query Strings: TS = ("Tai Chi" OR Taijiquan OR) AND TS = ("older adult*" OR elderly OR aging OR aged OR senior* OR geriatric*) AND TS = (health OR rehabilitation OR "fall prevention" OR balance OR cognition OR "health promotion")

Indices: SCI-EXPANDED, SSCI, A&HCI, ESCI.

Document Type: Articles and Review Articles.

Language: English.

Results: 1723 records.

3 Scopus

Query Strings: TITLE-ABS-KEY ("Tai Chi" OR taijiquan) AND TITLE-ABS-KEY ("older adult*" OR elderly OR aging OR aged OR senior* OR geriatric*) AND TITLE-ABS-KEY (health OR rehabilitation OR "fall prevention" OR balance OR cognition OR "health promotion")

Document Type: Article and Review.

Language: English.

Results: 1522 records.

4 Data Merging and Deduplication

Procedure: Records from both databases were exported in Plain Text (WoS) and RIS (Scopus) formats. The datasets were imported into **CiteSpace (v.6.4.R1)**, where the **"Remove Duplicates"** function was applied. A subsequent manual check of titles and DOIs was conducted to ensure data integrity, resulting in a final dataset of **2,532** unique publications.
